# Supplementary material for: Domain-specific schema reuse supports flexible learning to learn in the primate brain
Source: Nat Commun. 2026 Jan 29;17:2150. doi: 10.1038/s41467-026-68692-x (PMC12957312; doi:10.1038/s41467-026-68692-x)
Supplement: Supplementary file 1 — Supplementary Information [file 41467_2026_68692_MOESM1_ESM.pdf]

Supplementary Information

1. Supplementary Notes

1.1 Pre-trained Experience Influence

It is noteworthy that, before the recording, Monkeys AB and ZZ were trained using fixed visual stimulus pairs to familiarize them with the basic task structure (visual stimulus → button press). Then, along with the start of the electrophysiological recordings, the sequential learning paradigm, i.e., learning of multiple new stimulus pairs followed by reversal learning (A, B, Revisit-A, Reverse-A), was introduced to them. In contrast, Monkey XW received additional training on sequential learning of new visual stimulus – action pairs (A, B, C, Revisit A) prior to the start of electrophysiological recordings.

These pre-trained experiences did influence baseline learning speeds: behaviorally, Monkeys AB and ZZ generally required more trials to learn new stimulus-action mappings compared to Monkey XW. On average, monkeys required the following number of trials to reach the learning criterion for task A (mean ± SD): Monkey AB: 95.6 ± 38.2 trials per day; Monkey ZZ: 116.5 ± 37.25 trials per day; Monkey XW: 25.13 ± 8.79 trials per day. Despite differences in initial learning speed, all monkeys showed a consistent trend that once they had learned stimulus pair A, their subsequent learning of new pairs became faster, demonstrating a cross-task learning effect within a day (Fig. 2d). This consistency suggests that the observed acceleration in learning speed reflects the formation and utilization of schemas, rather than merely reflecting general task familiarity.

About the neural results, although Monkey XW showed more clear neural patterns in decision-related classification and activity, such as more distinct clustering of neural dynamics in the

early time period (Supplementary Fig. 10), all three monkeys demonstrated the same fundamental pattern of NCS in the decision subspace. This consistency in neural findings across animals with varying levels of prior experience provides evidence for the robustness of our key conclusions about domain-specific NCS formation and reuse.

1.2 Justification for Using LFADS Dimensionality Reduction Rather Than Raw Data for Classification

Neural spike data presents unique analytical challenges due to its high dimensionality, sparse structure, and inherent variability. Here, we explain our choice to apply dimensionality reduction using LFADS before CNN-based classification, rather than training classifiers directly on raw spike data.

To validate the LFADS approach, we conducted comparative decoding analyses using raw spike data, processed as binary time series sampled at 1 kHz with each bin indicating the presence (1) or absence (0) of spikes (Supplementary Fig. 2). Using the same classifier architecture as described in our manuscript, we observed clear evidence of overfitting when training directly on spike data. For example, when classifying decisions in Monkey ZZ, models trained on raw spikes rapidly achieved 100% accuracy on the training set while validation accuracy plateaued around 40% (Supplementary Fig. 2a). Similarly, the training loss quickly approached zero, while the validation loss gradually increased without convergence (Supplementary Fig. 2b). This divergence between training and validation performance is a signature of overfitting.

In addition, our quantitative comparison demonstrated that decoding performance with raw

spike data was substantially lower (Supplementary Fig. 2c). For Monkey XW, decoding based on raw spike data reached above-chance performance for motor decisions, but failed to classify visual stimuli effectively. For Monkeys AB and ZZ, spike-based decoding yielded near-chance performance for both tasks. These results were consistent across three days of recordings (error bars indicate mean ± SD).

This poor performance with raw spike data likely stems from several factors:

1. High trial-to-trial variability: Neural spike data exhibits substantial variability across trials, making it difficult to extract consistent patterns. This high-dimensional noise may obscure task-related signals, complicating direct classification[1].
2. Limited sample size: Neural datasets are high-dimensional but contain relatively few trials (~226 per day), increasing the risk of overfitting in neural network models[2]. In such cases, dimensionality reduction prior to classification can enhance robustness by isolating behaviorally relevant neural features while mitigating noise. Golub et al.[3] further demonstrated that low-dimensional neural representations effectively capture behaviorally relevant dynamics, even in constrained datasets, providing a more reliable basis for subsequent analyses.

LFADS provides a solution to these challenges by recovering smooth neural trajectories from noisy spiking data [4]. This method combines variational auto-encoders with recurrent neural networks to model neural population dynamics.

1.3 Rationale for Selecting 16 Dimensions in LFADS Analysis

To evaluate the impact of latent dimensionality in LFADS, we tested several settings (3, 6, 16, 26, and 36 dimensions) using data from monkey XW across three recording days. As shown in

Supplementary Fig. 3a, the reconstruction error of the spiking activity showed no significant differences across dimensionalities (one-way ANOVA, $P = 0.434$), suggesting that increasing the number of latent factors did not improve the model's ability to capture neural dynamics. We also evaluated the influence of dimensionality on downstream decoding performance. As shown in Supplementary Fig. 3b, classification accuracies for decision and stimulus conditions remained comparable across dimensions, with no consistent advantage at higher or lower settings. Error bars indicate standard deviation across the three sessions. Based on these analyses, we concluded that the specific choice of 16 dimensions does not critically affect our results and opted for this value as a representative latent dimensionality.

1.4 Single-Neuron Analyses of NCS

Understanding the single-neuron foundations underlying population-level NCS is critical for interpreting the neural mechanisms of flexible learning. While our primary analyses focused on population dynamics, here we characterize the tuning properties of individual PMd neurons to establish the cellular basis for the observed manifold reuse.

1.4.1 Functional Classification of PMd Neurons

We categorized all recorded PMd neurons based on their response properties using a two-way ANOVA (factors: stimulus identity and movement direction, $P < 0.01$), identifying five functional categories: Decision-selective neurons (cells significantly modulated by movement direction regardless of stimulus identity), Stimulus-selective neurons (cells significantly modulated by visual stimulus identity regardless of movement direction), Linear mixed-selectivity neurons (cells showing additive coding of both stimulus and decision variables), Non-linear mixed-selectivity neurons (cells showing interaction effects between stimulus and

decision variables), and Non-selective neurons (cells without significant tuning to task variables).

Supplementary Fig. 5a-c shows the distribution of neuronal response types from a representative recording day for each monkey. Consistent with previous reports of sparse task-variable tuning in prefrontal cortex [5], the majority of PMd neurons were non-selective to either task variable, suggesting that only a subset of neurons strongly contribute to the task-relevant population code.

1.4.2 Response Properties of Task-Selective Neurons

To illustrate the functional diversity in PMd, we computed trial-averaged peri-stimulus time histograms (PSTHs) for neurons with different selectivity patterns. All neural activity was aligned to stimulus onset (time = 0), with shaded areas representing ± SD. across trials (bin size = 200ms). Supplementary Fig. 6 shows representative examples of different neuronal types. Decision-selective neurons exhibited differential activity patterns for upper versus lower movement directions throughout the visual stimulus period (Supplementary Fig. 6). Stimulus-selective neurons demonstrated distinct temporal responses to different visual stimuli while showing similar activity patterns for the same motor outcome (Supplementary Fig. 6). Mixed-selectivity neurons were jointly modulated by both stimulus identity and movement direction, with complex response patterns that cannot be explained by either variable alone (Supplementary Fig. 6).

1.4.3 Population-Level Directional Preference

To evaluate whether intrinsic directional biases could account for the reuse of task-related neural representations across different learning tasks, we analyzed the population-level

preference of PMd neurons for upper versus lower target locations during the decision-making period, specifically from stimulus onset to the appearance of the response buttons. For each neuron, we computed a preference index following the method of Tan et al. [6], defined as：

$$\text{Preference Index} = \frac{FR_{upper} - FR_{lower}}{FR_{upper} + FR_{lower}} \tag{1}$$

Where FR_{upper} and FR_{lower} denote the mean firing rates for trials involving upper and lower button targets, respectively. This analysis was performed separately for Task A, Task B, and Task Revisit-A blocks within individual recording sessions.

Supplementary Fig. 7 illustrates the distributions of preference indices for representative recording sessions from each monkey. Across all three monkeys (XW, ZZ, and AB), we found no consistent directional bias at the population level. The distributions of preference indices were approximately centered around zero across tasks, with no significant directional preferences detected in most cases (Monkey XW: Task A, $P = 0.73$, mean = 0.012; Task B, $P = 0.65$, mean = –0.013; Task Revisit-A, $P = 0.40$, mean = –0.02; Monkey ZZ: Task A, $P = 0.073$, mean = 0.05; Task B, $P = 0.88$, mean = –0.004; Task Revisit-A, $P = 0.071$, mean = –0.09; two-sided t-tests). Only in Monkey AB during Task A was a mild significant preference toward the lower target observed ($P = 0.04$, mean = –0.07); however, no significant preferences were detected in Monkey AB's Task B ($P = 0.28$, mean = 0.04) or Revisit-A blocks ($P = 0.17$, mean = –0.06).

Furthermore, the distributions were unimodal rather than bimodal, indicating a continuous range of directional tuning rather than distinct neuronal subpopulations strongly favoring either the upper or lower targets. While occasional transient directional preferences emerged, they lacked consistency across tasks within a day and across monkeys. Therefore, it is unlikely that

the observed reuse of task-related neural dynamics across different visuomotor mappings is driven by pre-existing directional biases in PMd neuronal activity.

1.4.4 Stability of Neural Selectivity During Learning

To assess whether task-specific neural representations emerge as a function of learning, we compared neural selectivity between early (first 20% of trials) and late (last 20% of trials) phases of learning. Despite clear behavioral improvements (early phase: 80.64±3.70% correct; late phase: 93.01±2.15% correct), we found no significant differences in the neural representations. The proportions of decision-selective and stimulus-selective neurons did not significantly differ between early and late learning phases (decision: $P = 0.427$, Hedges' $g = 0.187$; stimulus: $P = 0.38$, $g = 0.436$; Supplementary Fig. 8). Additionally, Tuning Index (TI) values, which quantify the strength of selectivity for decision and stimulus variables[7], showed no consistent changes across learning (Supplementary Fig. 9).

These findings suggest that decision- and stimulus-related signals are already present in PMd prior to task mastery, and learning does not substantially recruit additional task-selective units or systematically strengthen existing selectivity. Rather, learning appears to involve reorganization of pre-existing neuronal ensembles without significant expansion of task-specific representations. This view aligns with recent findings by Drieu et al.[8], who demonstrated that rapid acquisition of latent task knowledge in sensory cortex occurs without substantial modification of pre-existing stimulus selectivity.

1.5 More Mathematical Details about dPCA

To extract low-dimensional neural representations associated with task-relevant variables, we

applied dPCA [9]. Unlike standard PCA, which finds global directions of maximum variance, dPCA separates population activity into components that are specifically aligned to task parameters (e.g., stimulus, decision) while preserving the temporal structure of the original data. Regarding the transformation method:

Our data matrix **X** (neurons × time points × conditions) is decomposed into parameter-specific marginalizations:

$$X = X_t + X_{\mathrm{st}} + X_{dt} + X_{sdt} + X_{noise} = \sum_{\phi} X_{\phi} + X_{noise} \tag{2}$$

Where X_t represents condition-independent activity, X_{st} captures stimulus-dependent variance, X_{dt} captures decision-dependent variance, and X_{sdt} captures stimulus-decision interactions. For each marginalization, dPCA finds decoder and encoder matrices (D_{ϕ} and F_{ϕ}) that minimize the reconstruction error between the marginalized data and the reconstructed version:

$$L_{dpca} = \sum_{\phi} \left\| X_{\phi} - F_{\phi} D_{\phi} X \right\|^2 \tag{3}$$

The components are then ranked according to explained variance to select the dimensions that capture the most prominent signals in the data. This approach is linear but differs from standard PCA in that decoder/encoder pairs for different task parameters are obtained independently from their respective marginalized data.

dPCA performs a factorial variance decomposition analogous to methods in classical statistics (e.g., Rutherford et al.[10]) to separate the stimulus and decision dimensions. For example, the marginalization for stimulus involves:

$$X_s(c) = \frac{1}{T} \sum_{t=1}^{T} X(c,t) - \bar{X} \tag{4}$$

$\bar{X}$ captured the mean neuronal responses across all conditions. This quantity represents the deviation from the overall mean that is attributable solely to stimulus factors. Similar calculations are performed for decision components and interaction components. Mathematically, this approach explicitly separates variance along different task parameters by defining these marginalized components before the dimensionality reduction step.

In applications, it is common to encounter the problem of imbalanced sample data. To mitigate imbalance issue (e.g., trials for the two button press directions were unequal under the same visual stimulus), we used the re-balanced dPCA formulation. As described in Kobak et al.[9], this modifies the loss function to treat all task parameter combinations equally, regardless of frequency:

$$L_{\phi} = \left\| \tilde{X}_{\phi} - FD\,\tilde{X} \right\|^2 + SQT \left\| FD\,C_{noise}^{1/2} \right\|^2 \quad (5)$$

Where $\tilde{X}$ represents mean firing rates (PSTHs) that can be collected in a smaller matrix of size $N \times SQT$. As noted in the paper: In the unbalanced case, we can directly use this last formulation where all occurrences of X have been replaced by $\tilde{X}$. This is especially useful for neural data, where some combinations of task parameters may occur more often than others. The 're-balanced' dPCA loss function treats all parameter combinations as equally important, independent of their occurrence frequency.

1.6 Rationale for Selecting 20 Dimensions in dPCA Analysis

The choice of dimensionality in dPCA represents an important methodological consideration that can influence the interpretation of neural data analyses. Here, we provide justification for our selection of 20 dimensions in the dPCA.

We evaluated the impact of dimensionality choice through multiple quantitative analyses across all three monkeys ($n = 3$ recording days per monkey). First, we quantified the total neural variance explained as a function of dimensionality (Supplementary Fig. 11a). At 20 dimensions, dPCA captured 66.98 ± 0.89% of variance for Monkey XW, 76.66 ± 3.07% for Monkey ZZ, and 86.24 ± 1.15% for Monkey AB (mean ± SD). To determine an objective cutoff point, we calculated the marginal gain in explained variance when adding dimensions (Supplementary Fig. 11b). The average marginal gain across dimensions 20–25 was 3.92 ± 1.20%, falling below our empirically determined 5% threshold, indicating diminishing returns beyond this point.

We next examined how component distribution varies with dimensionality. Supplementary Fig. 11c shows the proportion of dimensions allocated to different component types (stimulus, decision, and others) across dimensionality settings (10–35). While higher dimensionality slightly increases the absolute number of stimulus- and decision-related dimensions, even at lower settings (e.g., 10 dimensions), the analysis reliably identifies at least one decision component and four stimulus components across all animals. This stability reflects the dPCA computational framework, where neural data are marginalized into different components with dimensionality reduction performed separately on each component. Consequently, increasing dimensions does not alter previously selected components but adds new ones based on variance ranking.

The near-orthogonal relationship between stimulus- and decision-related subspaces across different dimensionality settings provides further validation for our choice (Supplementary Fig. 11e). At 10 dimensions, the angular separation between these subspaces does not significantly differ from shuffled controls, suggesting insufficient information capture for accurate

representation of near-orthogonal structures. As dimensionality increases, the near-orthogonality angles stabilize in both monkeys, with 20 dimensions providing an optimal balance between information retention and statistical differentiation from shuffled conditions. Higher dimensionalities (e.g., 30) maintain stable near-orthogonality angles but do not significantly enhance discriminability and potentially introduce additional noise.

These analyses indicate that our choice of 20 dimensions achieves a balance between capturing sufficient neural variance and ensuring computational tractability. Additionally, our key findings regarding domain-specific patterns, such as representation and reuse, as well as the approximate orthogonality between the decision- and stimulus-related subspaces, remain robust to this choice.

1.7 Validation of Subspace Near-Orthogonality Through Selective Label Shuffling Controls

To validate that the observed near-orthogonal relationship between stimulus and decision subspaces reflects genuine neural organization rather than methodological artifacts, we performed targeted control analyses with selective label shuffling. In our primary analysis, we compared the actual subspace angles to those obtained after randomly shuffling both stimulus and decision labels for each trial. This shuffling approach removes all relationships between neural activity and task variables, providing a stringent null distribution. However, to address the possibility that simultaneous shuffling of both labels might artificially disrupt any existing near-orthogonality, we conducted two additional selective shuffling control analyses:

1. Decision-only shuffling: We maintained the original stimulus label assignments while

randomly permuting only the decision labels across trials (Supplementary Fig. 14a).

2. Stimulus-only shuffling: We preserved the original decision label assignments while randomly permuting only the stimulus labels across trials (Supplementary Fig. 14b).

Both selective shuffling controls produced subspace angles that were significantly smaller than those in the original data in monkey AB and XW, while monkey ZZ also showed a trend toward orthogonality, and both cross-animal analyses showed significant reductions. This is particularly informative because if the observed near-orthogonality were merely a methodological artifact of the dPCA procedure, selectively shuffling only one variable type would not reduce the angular separation between subspaces.

These results provide compelling evidence that the observed near-orthogonality between stimulus and decision subspaces reflects genuine segregation of information within the neural population rather than an artifact of our analytical approach. The fact that any controlled disruption of the true label structure diminishes the angles indicates that PMd neural populations actively maintain separable representations of stimulus and choice information through approximately orthogonal encoding schemes.

1.8 Terminological Clarification – Schema vs. Neural Correlates of Schema (NCS)

The concept of a schema originates from cognitive psychology and refers to structured mental frameworks that support the assimilation of new information and guide future behavior [11,12,13]. These constructs are generally studied at the behavioral level and are considered to emerge from distributed neural systems.

When neuroscientists investigate schemas at the neural level, they typically study the neural

instantiation or neural correlates of schema-related processes. Recent neuroscience literature has made significant progress in identifying neural mechanisms underlying schema functions [14,15]. However, there has not been consistent terminological differentiation between the cognitive concept and its neural implementation across the neuroscience literature. This lack of distinction can potentially lead to imprecise claims about the relationship between measured neural activity and the broader cognitive construct.

We use neural correlates of schema (NCS) to indicate neural population activity patterns that exhibit computational properties associated with schemas—namely, stability across similar contexts, transferability to new but related situations, and facilitation of subsequent learning. These neural representations reflect structured, task-general patterns that support flexible learning, but we do not equate them with the full cognitive construct of a schema.

This distinction is important because cognitive schemas likely emerge from distributed neural systems that extend beyond what we measured in the dorsal premotor cortex (PMd). While our recordings reveal neural activity patterns that show schema-related properties, we make no claim that these patterns constitute a complete neural instantiation of a cognitive schema. Our approach aligns with established terminology in neuroscience literature [16,17,18].

1.9 Understanding Separable but Unstable Neural Representations in Stimulus-Related Subspaces

Our analyses revealed an unexpected instability in PMd stimulus-related neural representations between Task A and Revisit-A, even though visual stimuli and sensorimotor mappings were identical. This phenomenon, which can be understood as separable but unstable dynamic

encoding, requires careful clarification.

First, we clarify that we use the term stimulus-related subspace in our text to describe variance components associated with stimulus processing, rather than implying that PMd stably encodes physical stimulus features. This distinction is critical, given PMd's established role in transforming sensory inputs into action-relevant representations, rather than preserving veridical sensory identities. Separability here refers to the ability to distinguish different conditions, reflected in distinct patterns of neural activity. Stability refers to the consistency of neural representations for the same condition across different times (e.g., A vs. Revisit A). These two concepts are not contradictory, as separability operates within conditions while stability operates across conditions.

Prior studies have shown that PMd integrates sensory information in a context-dependent manner to guide motor planning. Pesaran et al.[19] reported that PMd activity preceded that of the parietal reach region during free-choice tasks, but only when decisions were required, suggesting rapid transformation of visual inputs for decision-making. Similarly, Hoshi and Tanji [20] demonstrated that PMd maintains sensory evidence tied to behavioral goals rather than encoding static visual features. Anatomically, PMd receives multimodal inputs from prefrontal and parietal cortices, consistent with its role in context-dependent sensorimotor integration.

In our case, the separable but unstable representations may reflect context-dependent dynamic remapping of stimulus information in PMd. Visual stimuli in tasks A, B, and C can be distinguished within their contexts, but following new learning experiences and contextual influences, the same visual stimuli become difficult to distinguish and unstable between tasks

A and Revisit-A. This suggests that stimulus-related information representations are influenced by contextual factors, leading to dynamic reorganization of the encoding space while preserving within-context discriminability.

To better understand this separable but unstable dynamic coding approach, we constructed a linear toy model of dual-context classification tasks to elucidate dynamic encoding, demonstrating the possible mechanisms underlying the coexistence of separability and instability. We defined two stimulus templates $V_1 = [1.0,0]^T$ and $V_2 = [-0.6,0.4]^T$, and implemented context-dependent encoding through linear transformation matrices: identity transformation $T_0 = I$ (context 0) and rotation transformation $T_1 = R(\theta)$ (context 1), where $R(\theta) = [cos\theta\ sin\theta;\ -sin\theta\ cos\theta]$, $\theta = 10°$. The data generation process was: $X_{c.i} = T_c V_i + \varepsilon$, where $\varepsilon \sim N(0, \sigma^2 I)$, $\sigma = 0.25$. As shown in Supplementary Fig. 17, the left panel displays the representational patterns of two stimulus categories in context 0, with dark blue circles and dark red triangles representing the response distributions of the two stimulus types, exhibiting clear separability. The right panel shows the same stimulus categories in context 1, marked with light colors, which maintain within-class clustering and between-class separation after 10° rotation transformation.

This toy model demonstrates the coexistence of separability and instability: (1) Within-context separability: within each context, the two stimulus categories can be effectively distinguished; (2) Cross-context instability: classifiers trained in context 0 show degraded performance in context 1, reflecting dynamic reorganization of neural representations. The above content demonstrates that characteristics that are both separable and unstable can exist, and stimulus-related subspaces exhibit precisely such features.

1.10 Discussion of Near-Orthogonal Relationship Between Visual Stimulus-Related Subspace and Decision Subspace

Our findings reveal that the stimulus-related subspace exhibits instability while the decision subspace contains relatively stable neural cognitive schemas (NCS). This raises a fundamental question that how an unstable subspace (stimulus-related) can maintain near-orthogonality with a stable subspace (decision-related).

To illustrate this phenomenon, we propose a geometric analogy. Consider the stable decision subspace as existing within a fixed manifold in the xy-plane of 3D neural activity space, while the unstable stimulus-related subspace operates along the z-axis without constrained manifold structure, allowing dynamic representational transformations. Crucially, regardless of how neural activity along the z-axis transforms or reorganizes, it does not affect the geometric orthogonality with the xy-plane (see Supplementary Fig. 18). This demonstrates that instability within a subspace does not preclude near-orthogonality with another subspace. The fundamental principle is that unstable transformations occur within a dimensionally constrained space that maintains its near-orthogonal relationship with the stable subspace. The instability reflects dynamic reconfiguration of stimulus-related representations to accommodate changing task contexts. This architectural principle enables the neural system to simultaneously maintain stable decision-related computations while flexibly adapting stimulus-related processing strategies within the same cortical region, supporting the coexistence of cognitive stability and adaptive flexibility.

1.11 Discussion of NCS in Reversal Learning

A question is whether the behavioral deficits observed in reversal learning reflect interference from established schemas or simply represent challenges in reversing specific sensorimotor mappings. This distinction is crucial for understanding the nature of cognitive flexibility and schema utilization.

To address this question, we examined neural manifold representations across different stages of reversal learning. Using monkey ZZ as an example (the monkey who successfully learned the reversal task), we compared decision manifolds formed during early reversal learning (first 20 trials), late-stage reversal learning (after reaching criterion performance), and the original Task A (Supplementary Fig. 19). Our analysis revealed that both early and post-learning reversal manifolds differed substantially from the Task A manifold. Critically, if reversal learning simply involved relearning visual-motor mappings while utilizing the same NCS, we would expect the learned reversal manifold to resemble the original Task A manifold. However, our results show no evidence of manifold reuse across learning stages. Instead, reversal learning involved the formation of entirely new decision manifolds, distinct from those supporting continuous learning tasks. This finding suggests that reversal learning requires constructing new NCS rather than simply flipping existing mappings within established schemas. The inability to reuse existing decision representations indicates that the cognitive demands of reversal tasks fundamentally differ from those of similar forward-learning tasks.

This suggests that while humans can readily understand abstract rules of associative learning tasks and transfer them to reversal contexts, monkeys may employ different strategies. Behavioral performance in reversal tasks shows considerable variability across individuals and

species [21, 22]. This indicates that the NCS observed in our study optimized for generalization across similar tasks, but their utility may be constrained when task demands fundamentally violate learned structural assumptions, necessitating the construction of entirely new representational frameworks.

1.12 Discussion of Near-Orthogonal Neural Encoding

Our study identifies near-orthogonal rather than perfectly orthogonal relationships between stimulus-related and decision-related subspaces in PMd. This distinction is crucial for understanding the biological constraints and computational advantages of neural encoding strategies. Perfect orthogonality implies zero correlation, which is theoretically unlikely in associative learning tasks where both subspaces participate in the learning process and stimuli guide decision formation to some extent. The primary goal of our study is not to prove absolute orthogonality, but rather to demonstrate how the neural system achieves effective separation of stimulus-related and decision information through near-orthogonal encoding. This structure provides computational advantages over random encoding by minimizing cross-dimensional interference while maintaining necessary information interactions, representing an optimal compromise between information separation and integration in associative learning contexts. Therefore, we have adopted the term near-orthogonal throughout the manuscript to accurately describe this phenomenon.

2. Supplementary Figures

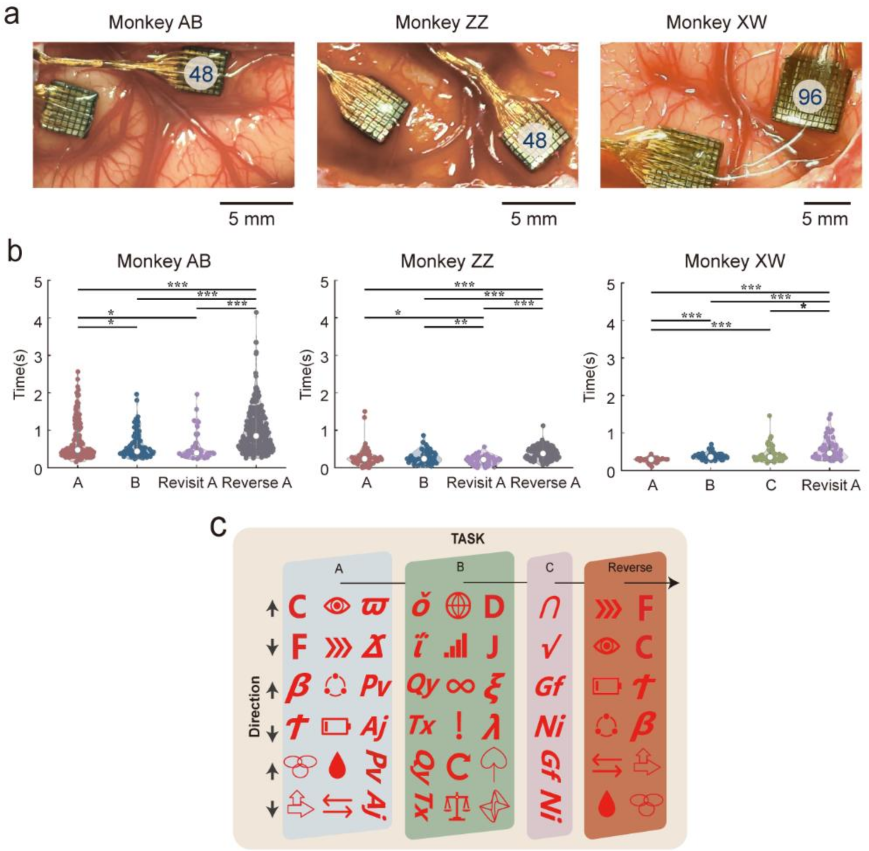

Supplementary Fig. 1. Electrode implantation and response time across tasks. (a) UTAH electrode implantation sites in three monkeys. All arrays were implanted in the left hemisphere. (b) Violin plots of response times (interval between the Go cue and hand release) across different learning tasks. Each dot represents the response time of an individual trial recorded on a given day. Violin plots show the distribution of data; the white dot indicates the median. (Monkey AB: A vs. B: $P = 0.04$, $g = 0.28$, A vs. Revisit-A: P=0.03, $g = 0.35$, A vs. Reverse-A: $P = 0.00003$, $g = -0.42$, B vs. Reverse-A: P<0.001, $g = -0.69$, Revisit-A vs. Reverse-A: P<0.001, $g = -0.749$; Monkey ZZ: A vs. Revisit-A: $P = 0.043$, $g = 0.371$, A vs. Reverse-A: $P = 0.0001$, $g = -0.56$, B vs. Revisit-A: $P = 0.006$, $g = 0.55$, B vs. Reverse-A: $P = 0.0002$, $g = -0.61$, Revisit-A vs. Reverse-A: P<0.001, g=-1.19; Monkey XW: A vs. B: $P < 0.001$, $g = -1.25$, A vs. C: P<0.001, $g = -0.92$, A vs. Revisit-A: P<0.001, $g = -1.31$, B vs. Revisit-A: $P = 0.00003$, g=-0.84, C vs. Revisit-A: P=0.01, $g = -0.51$).*$P < 0.05$, **$P < 0.01$, ***$P < 0.001$, two-tailed paired t-test. (c) The full set of visual stimuli.

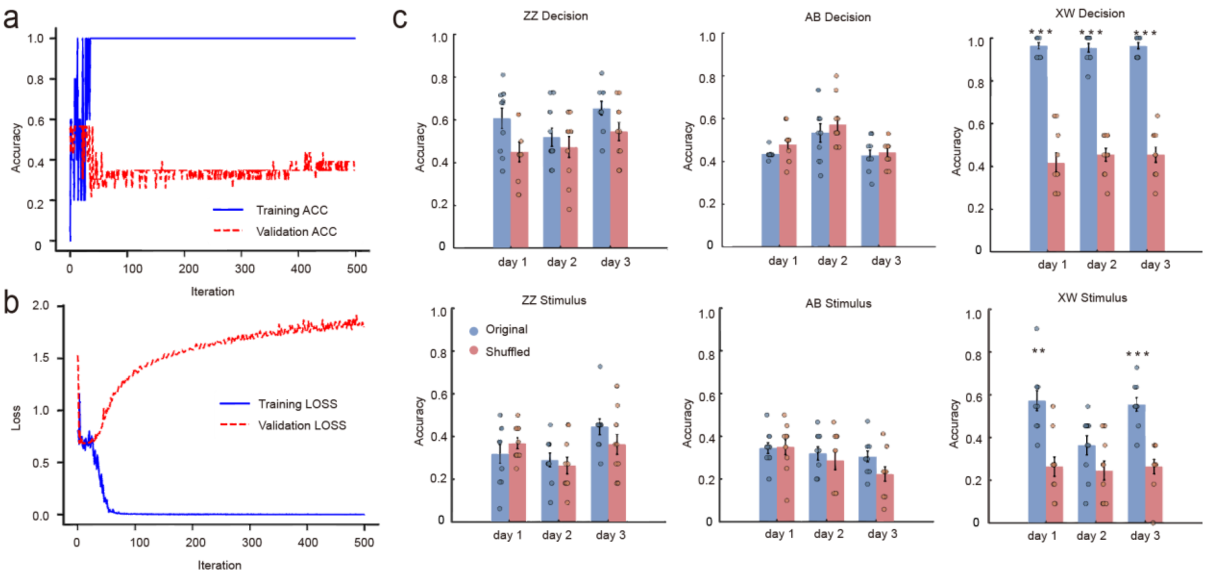

Supplementary Fig. 2. Training CNN classifiers on raw spike data for decision and stimulus classification. (a) Training accuracy (solid blue) and validation accuracy (dashed red) curves over iterations for a decision classifier trained on raw spike data from one recording day of monkey ZZ; (b) Training loss (solid blue) and validation loss (dashed red) curves over iterations for the same decision classifier as in panel a; (c) Decoding performance comparison between raw spike data (blue) and shuffled controls (red) across all monkeys. Upper panels show decision classification accuracy and lower panels show stimulus classification accuracy, presented as mean classification accuracy (±SD) across three recording days. (n = 3 sessions; XW stimulus: day 1: P = 0.002, Hedges' g = 2.02, day 3: P = 0.0009, g=2.67; XW decision: day1: P <0.001, g = 4.90, day2: P<0.001, g = 5.87, day 3: P <0.001, g = 5.62).*, P<0.05, **, P<0.01, ***, P<0.001, two-tailed paired t-test.

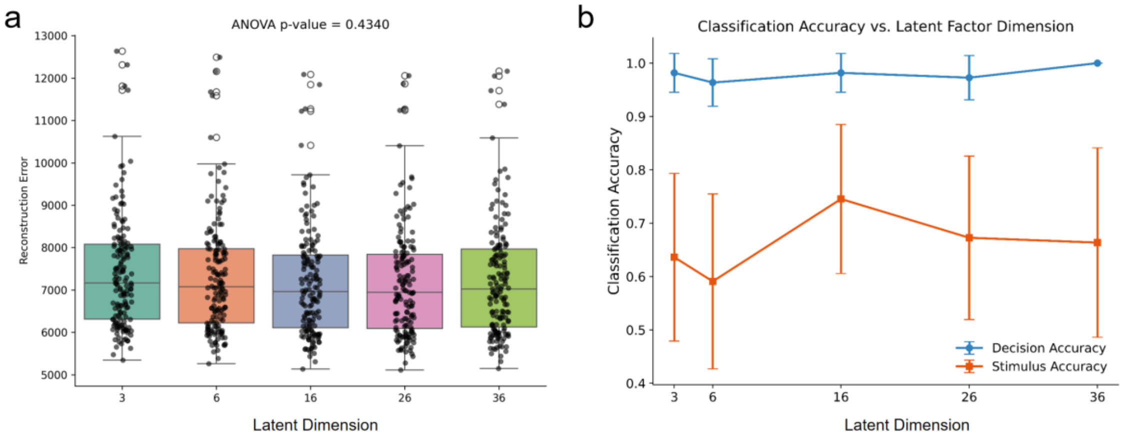

Supplementary Fig. 3. Effects of LFADS dimensionality on reconstruction error and decoding performance. (a) Reconstruction error of neural activity under different LFADS latent dimensionalities (3, 6, 16, 26, and 36), computed as mean squared error between original and reconstructed spike trains (monkey XW, single session, one-way ANOVA, p = 0.43). (b) Decoding performance for decision (blue) and stimulus (orange) variables based on latent trajectories from LFADS models of varying dimensionality. Bars indicate mean classification accuracy across three sessions from monkey XW; Error bars denote SD.(n = 3 sessions).

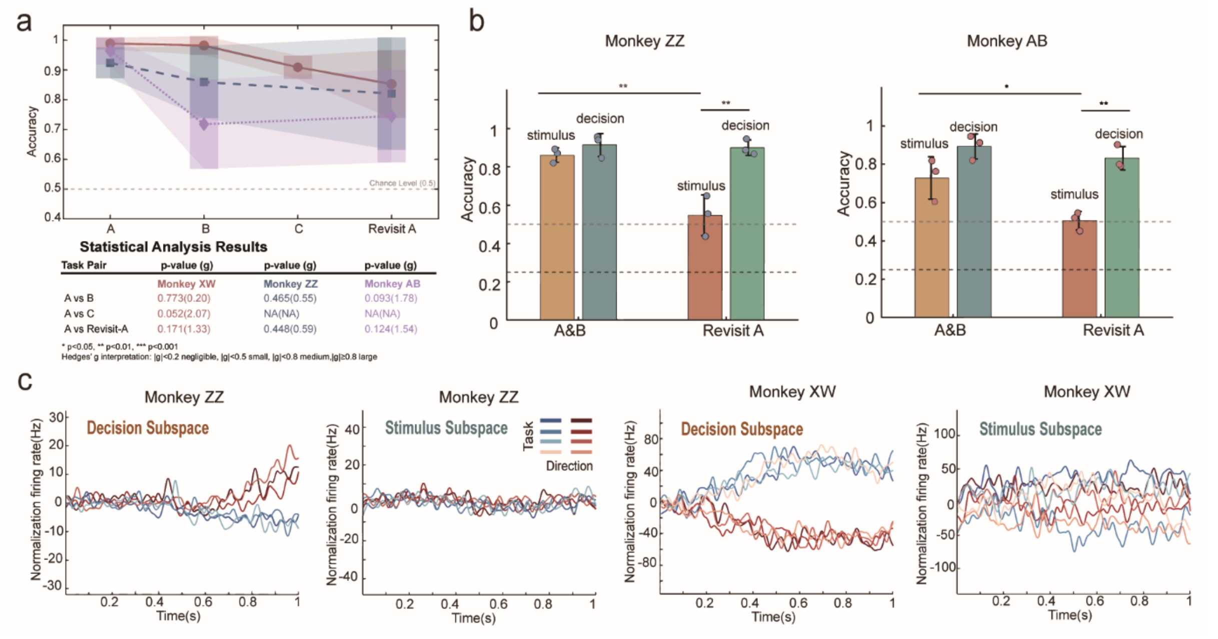

Supplementary Fig. 4. Cross-task generalization of decision decoders and neural subspace decomposition. (a) Generalization performance of decision decoders across tasks in individual monkeys. A decoder trained to classify decisions in Task A was tested on Tasks B, C, and revisit-A. Each line represents a monkey (*n* = 3 sessions per monkey); shaded areas denote across-day standard deviation. Statistical comparisons were performed using two-tailed t-tests with Hedges' g as the effect size. (b) Classifiers trained on Task A and B in two monkeys were used to decode visual stimuli and motor decisions and generalized to the revisit A task. The dark gray dashed line represents the chance level for visual stimulus classification (0.25), while the light gray dashed line indicates the chance level for decision classification (0.5). Colored scatter dots represent individual sessions. (n = 3 sessions per monkey, Monkey ZZ: stimulus A&B vs. Revisit-A: P =0.008, g=3.96; Revisit-A: stimulus vs. decision: P=0.006, g=-4.40; Monkey AB: stimulus A&B vs. Revisit-A: P = 0.03, g = 2.61; Revisit-A: stimulus vs. decision: P = 0.002, g = -5.85). *, $P < 0.05$, **, $P < 0.01$, ***, $P < 0.001$, two-tailed paired t-test. (c) Decompose neural population activity into the decision and the stimulus-related subspace for monkey ZZ and monkey XW for a representative session. Similar results were obtained across 3 sessions per monkey.

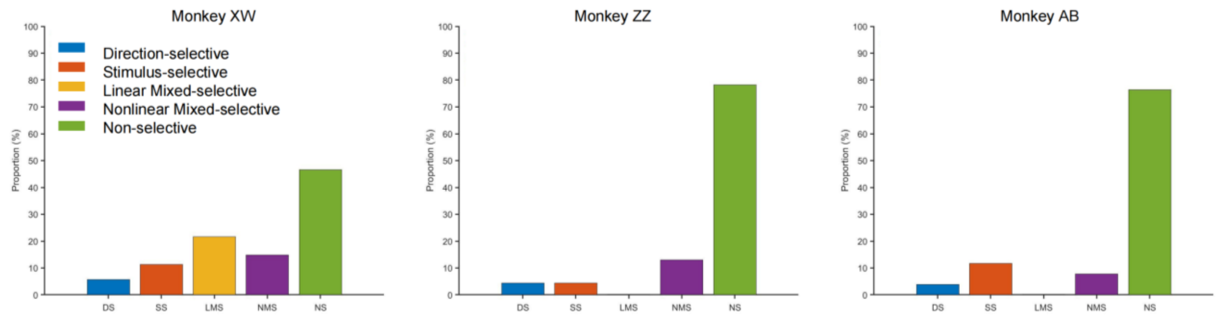

Supplementary Fig. 5. Proportion of functionally distinct neuron types across monkeys. Distribution of neuronal response types in monkey XW , ZZ , and AB, shown from one representative recording day per monkey. Neurons were classified using two-way ANOVA (factors: stimulus identity and movement direction) with significance threshold of $P < 0.01$.

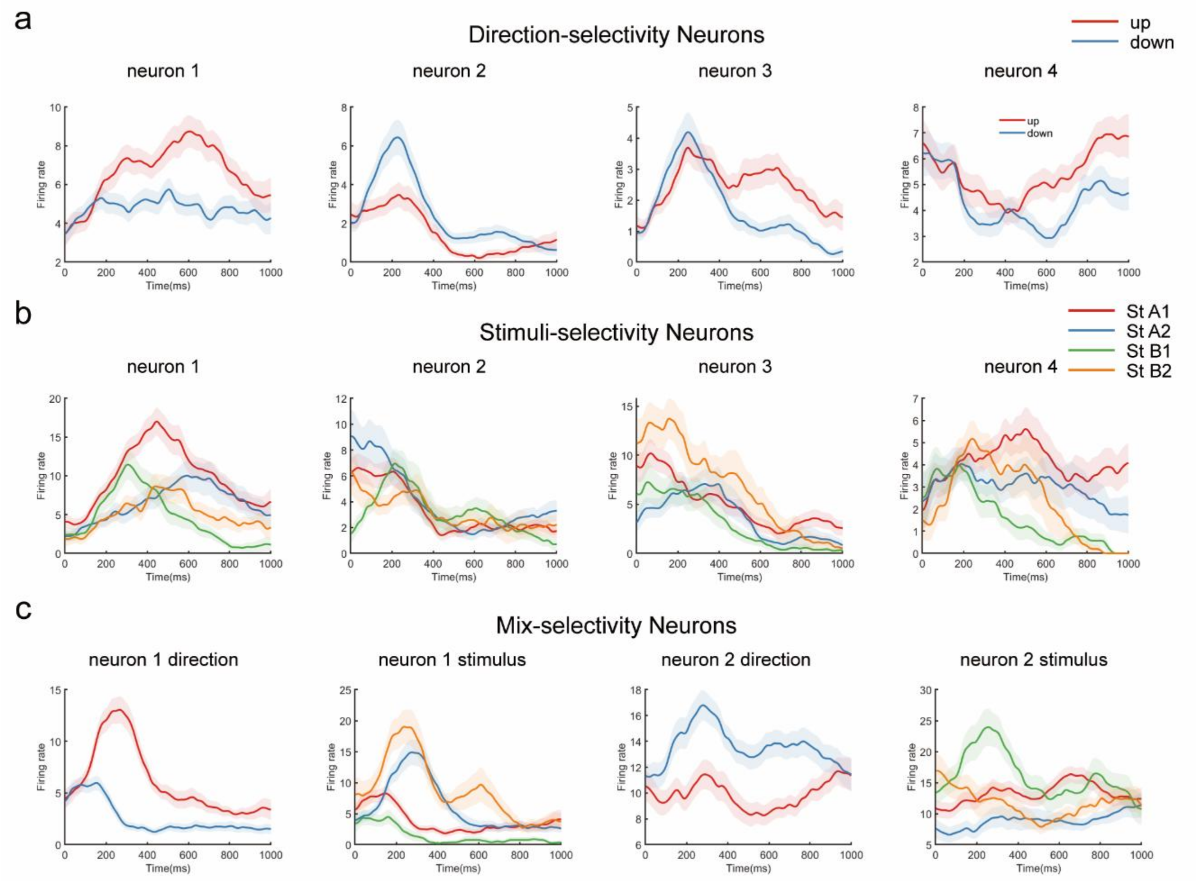

Supplementary Fig. 6. Examples of single-neuron tuning in PMd. Trial-averaged peri-stimulus time histograms (PSTHs) of representative PMd neurons recorded during the visuomotor mapping task. Neural activity is aligned to stimulus onset (time = 0). Shaded areas indicate the standard error of the mean (SD) across trials. (a) Decision-selective neurons showing differential activity for upper vs. lower button presses. (b) Stimulus-selective neurons exhibiting distinct responses to different visual stimulus. (c) Mixed-selectivity neurons modulated jointly by both stimulus identity and movement direction. St A1, Stimulus A1; St A2, Stimulus A2; St B1, Stimulus B1; St B2, Stimulus B2.

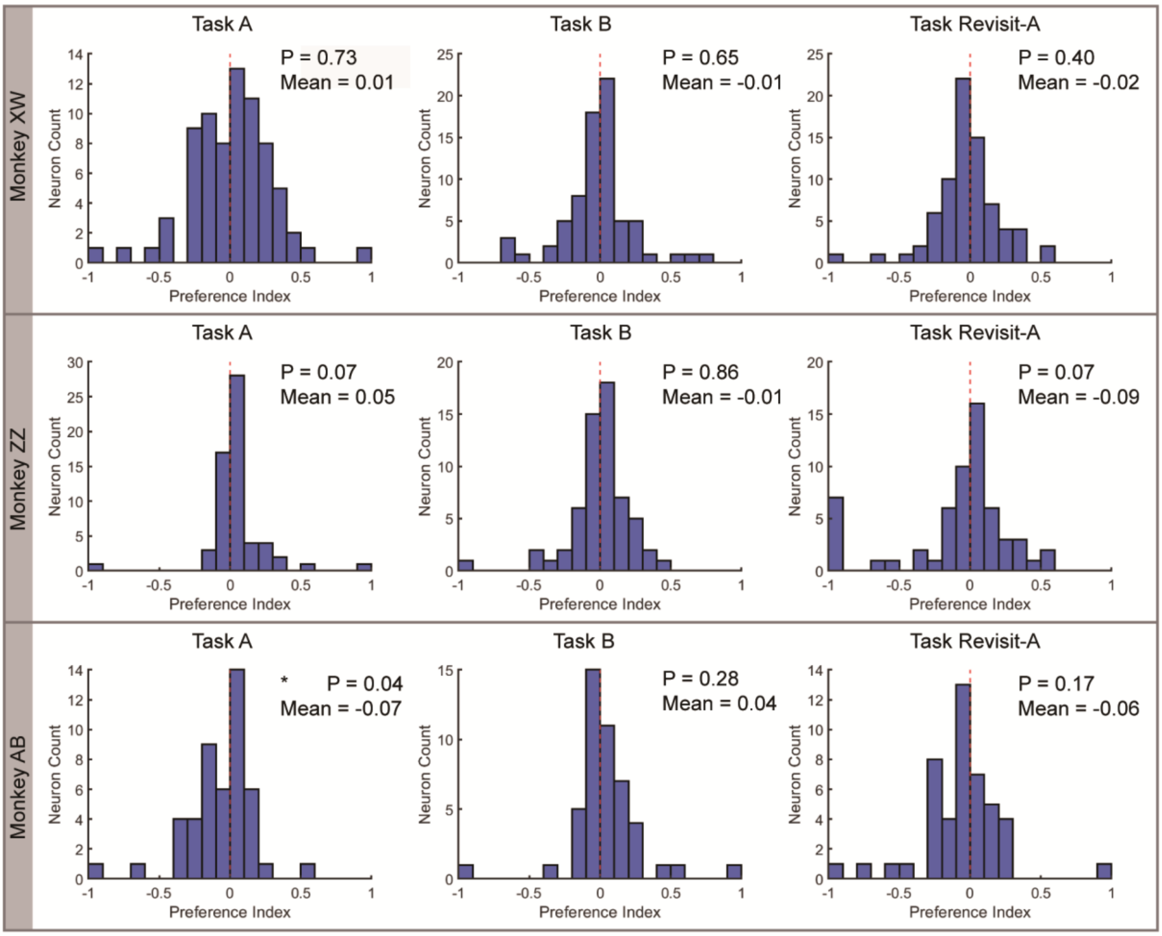

Supplementary Fig. 7. Population preference indices for upper versus lower response buttons in different tasks within one day in three monkeys for a representative session. Each bar plot shows the number of neurons exhibiting different preference index values, with positive values indicating preference for upper button responses and negative values indicating preference for lower button responses. Each column represents one task. Colored vertical lines indicate the mean preference index for each task. * denote significant deviation from zero (*, $P < 0.05$, **, $P < 0.01$, ***, $P < 0.001$; two-tailed t-test; Mean value indicates the average preference index for the task).

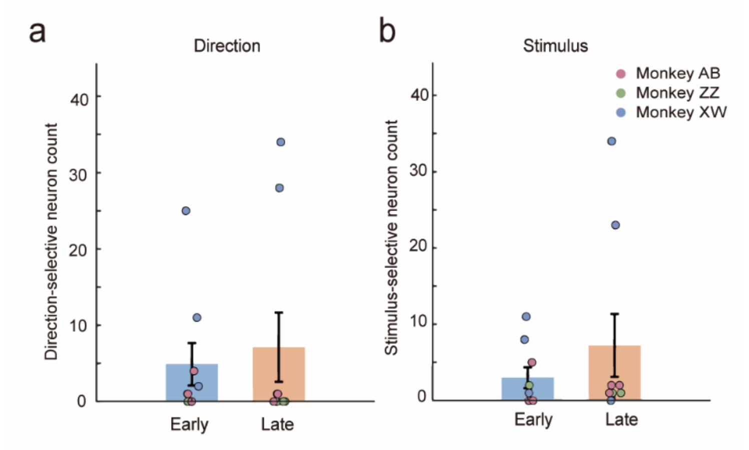

Supplementary Fig. 8. Neurons counts of task-selective PMd neurons in early and late learning phase. (a) Bar plots showing the mean ± SD proportion of direction-selective neurons in the early (blue) and late (orange) learning phases across three monkeys, n = 9 sessions. (b) Bar plots showing the mean ± SD proportion of stimulus-selective neurons in the early (blue) and late (orange) phases, n = 9 sessions. Colored scatter dots represent individual sessions, with each color corresponding to one monkey.

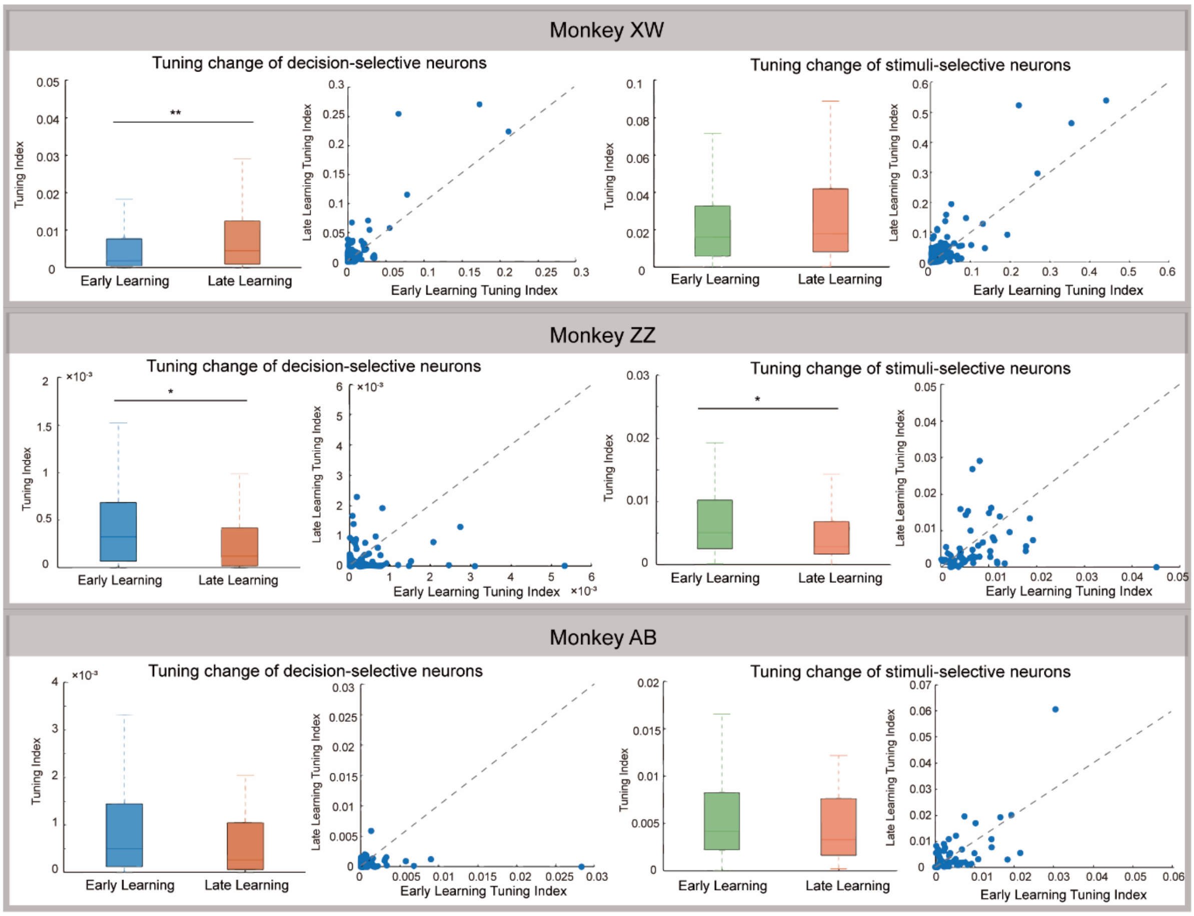

Supplementary Fig. 9. Changes in single-neuron TI across learning. Each row shows data from a representative recording day for one monkey, displaying box-and-whisker plots and corresponding scatter plots for decision (left column) and stimulus (right column) selectivity TI, comparing early (blue) and late (orange) learning phases. In box plots, the center line indicates the median; box edges represent the first (Q1) and third (Q3) quartiles; whiskers extend to the full data range. Scatter plots show individual neuron comparisons of TI before versus after learning. Each point represents one neuron; the unity line (dashed) indicates no change in selectivity. Points above the unity line denote neurons that became more selective, and points below denote neurons that became less selective. (Monkey XW: n =132 neurons; decision-selective: P = 0.001, g = -0.19; Monkey ZZ: n =61 neurons; decision-selective: P =0.046, g =0.32; stimulus-selective: P = 0.03, g = 0.24). *, $P < 0.05$, **, $P < 0.01$, ***, $P < 0.001$; two-tailed t-test.

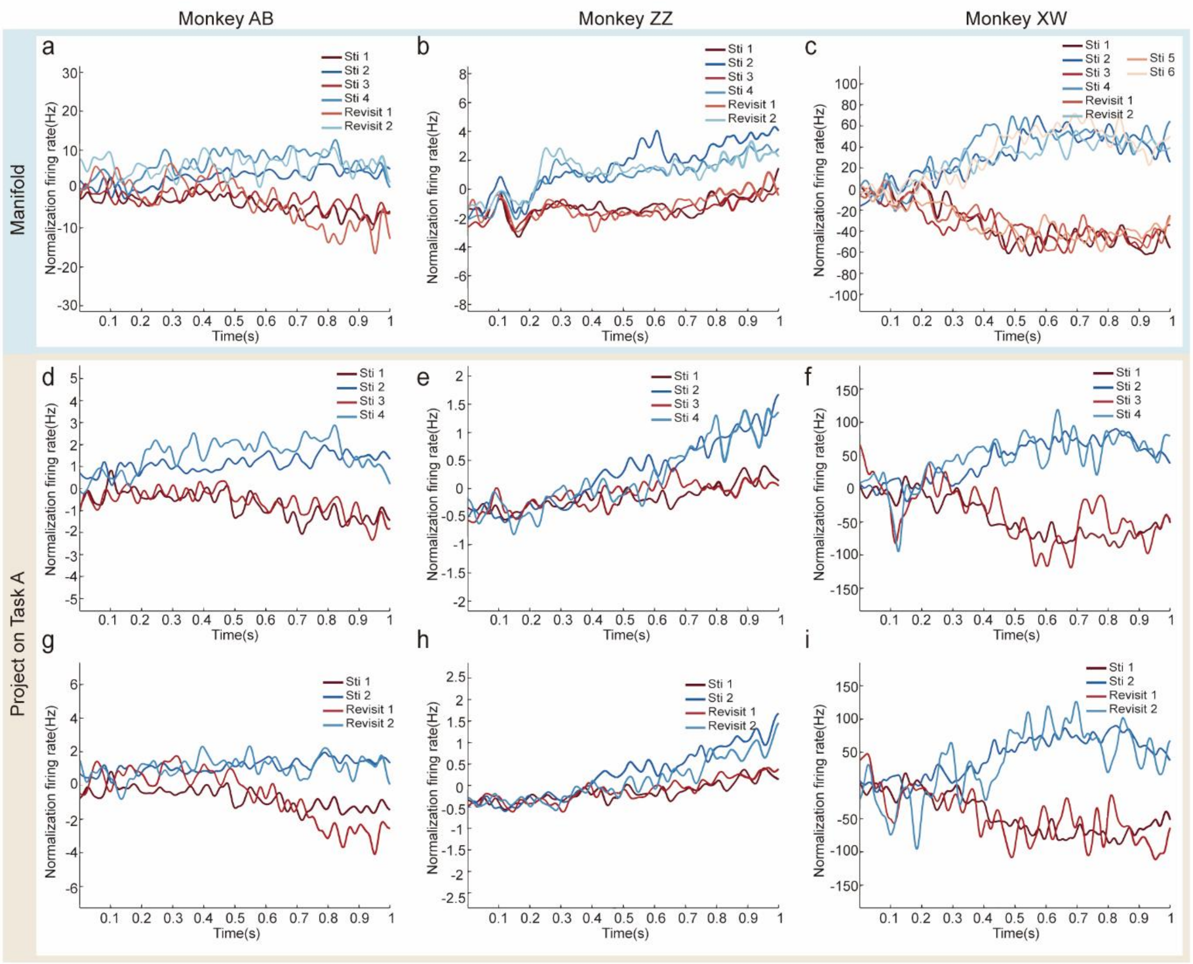

Supplementary Fig. 10. Decision-related subspace manifolds in three monkeys for a representative session. (a, b, c) Neural activities from different tasks, including Tasks A, B/C, and Revisit A, were projected onto a common decision subspace for each of the three monkeys. (d, e, f, g, h, i) To verify whether the manifold formed in Task A was reused in subsequent tasks; (d, e, f) Neural activity from Task B was projected onto the same decision subspace of Task A; (g, h, i) Neural activity from the Revisit A task was projected onto the same decision subspace of Task A. Sti, stimulus.

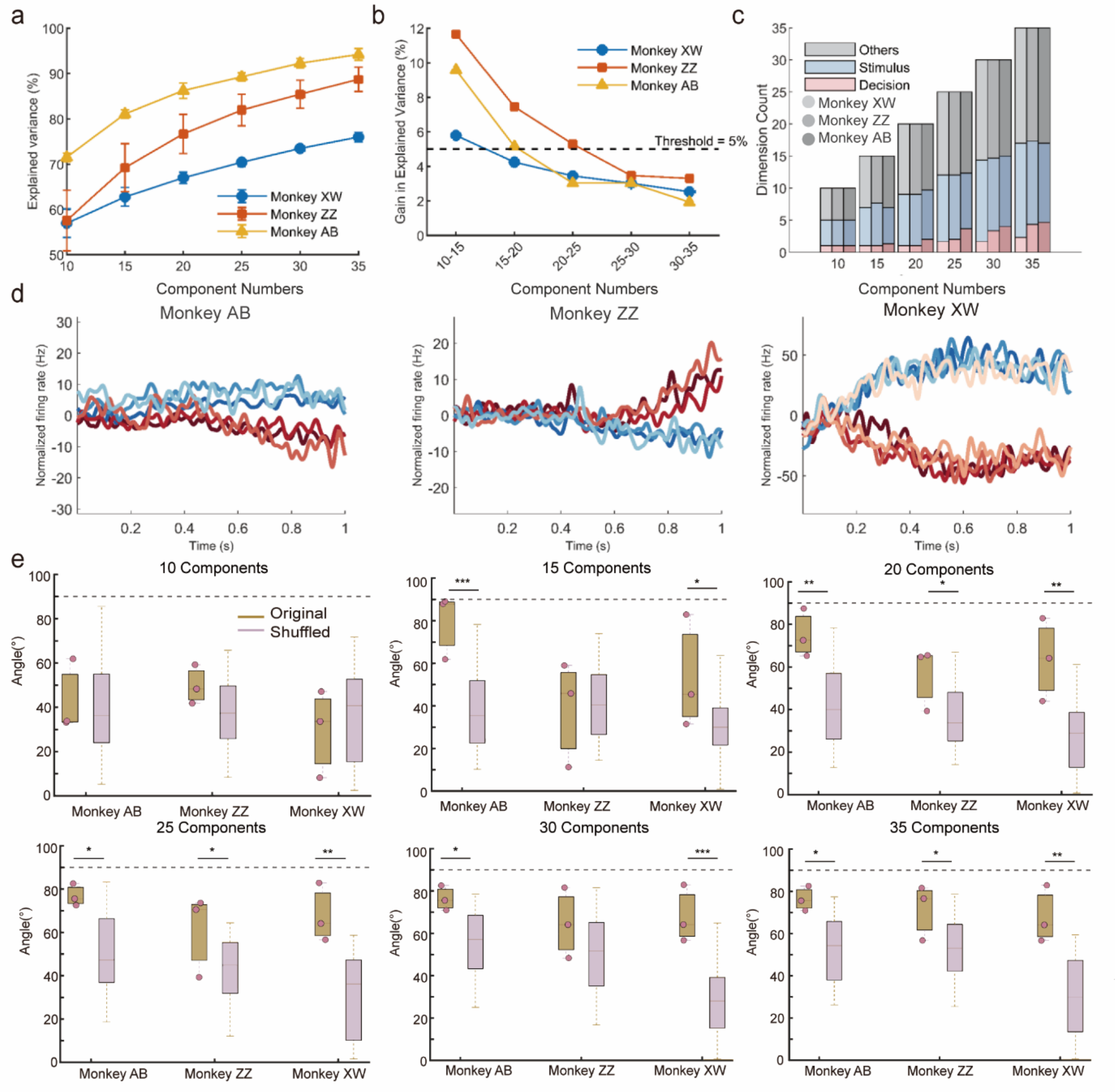

Supplementary Fig. 11. Analysis of dPCA dimensionality effects on neural representation. (a) Cumulative explained neural variance as a function of dPCA dimensionality. Each curve represents data from one monkey (XW, AB, ZZ), averaged across three recording days (n = 3 per monkey). Error bars indicate standard error of mean (SD); (b) Marginal gain in explained variance with increasing dimensionality. Each data point shows the additional variance explained when adding five dimensions. Horizontal dashed line indicates the 5% threshold used as criterion for dimensionality selection. Error bars represent SD across three recording days per monkey (n = 3 per monkey); (c) Component distribution analysis across dimensionality settings. Stacked bars show the relative proportion of dimensions allocated to stimulus encoding (blue), decision encoding (red), and other components (gray, including time and interaction effects) at different dPCA dimensionalities. Each group of three bars represents data from the three monkeys, with heights indicating mean values across three recording sessions per monkey (n = 3 per monkey); (d) Representative decision-related neural trajectories at 10-dimensional dPCA. Traces show neural activity projected onto the primary decision component, with upper button choices in red and lower button choices in blue. Different shading intensities represent different stimulus conditions. Each panel shows data from one representative recording session per monkey; (e) Angle between stimulus and decision subspaces across dimensionality settings. Data are shown as box-and-whisker plots. The line within each box indicates the median; box edges represent the first (Q1) and third (Q3) quartiles; whiskers show the full data range. Colored scatter dots represent individual sessions. (Original group: n = 3 per monkey; Shuffled group: n = 30 per monkey; 15 components: Monkey AB: P = 0.0009, g = 2.16; Monkey XW: P=0.04, g=1.34; 20 components: Monkey AB: P=0.005, g = 1.77; Monkey ZZ: P = 0.04, g = 1.27; Monkey XW: P = 0.002, g = 2.02; 25 components: Monkey AB: P = 0.02, g = 1.39; Monkey ZZ: P = 0.05, g = 1.23; Monkey XW: P = 0.003, g = 1.91; 30 components: Monkey AB: P = 0.02, g = 1.39; Monkey XW: P = 0.0007, g=2.23; 35 components: Monkey AB: P = 0.01, g =1.54; Monkey ZZ: P = 0.037, g = 1.29; Monkey XW: P=0.002, g=2.09). *, P<0.05, **, P<0.01, ***, P<0.001, Watson-Williams test.

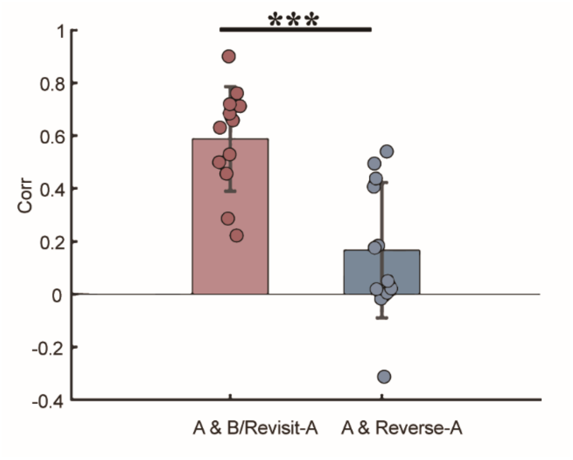

Supplementary Fig. 12. Principal component representations of decision-related neural subspaces across Tasks A, B, Revisit-A, and Reverse-A, pooled across two monkeys. Error bars indicate standard deviation across six recording sessions (n = 3 sessions per monkey). Each dot represents the similarity for one direction (up or down) manifold within a recording session, yielding twelve data points in total. (P = 0.0002, g = 1.78). *, $P < 0.05$; **, $P < 0.01$; ***, $P < 0.001$; two-tailed t-tests.

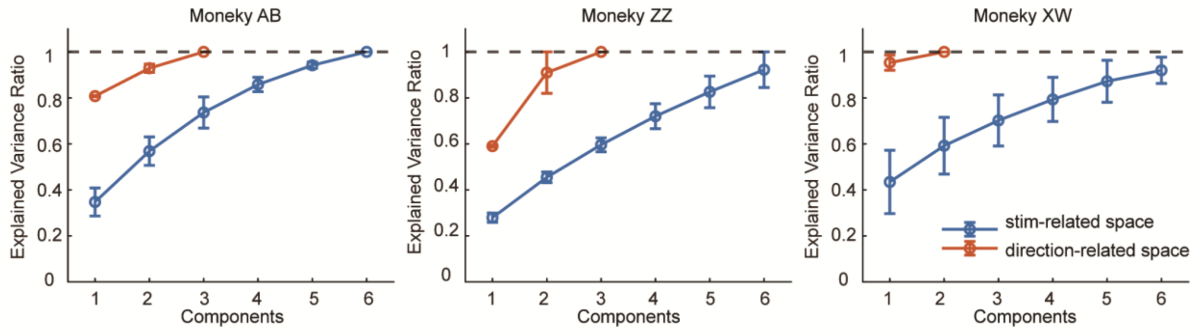

Supplementary Fig. 13. Cumulative variance explained by each dimension in the decision and stimulus-related subspaces. For each monkey, the cumulative variance explained by individual dimensions is shown for the decision (orange) and stimulus (blue) subspaces. Data are presented as mean ± SD, averaged across 3 recording days (n = 3 sessions).

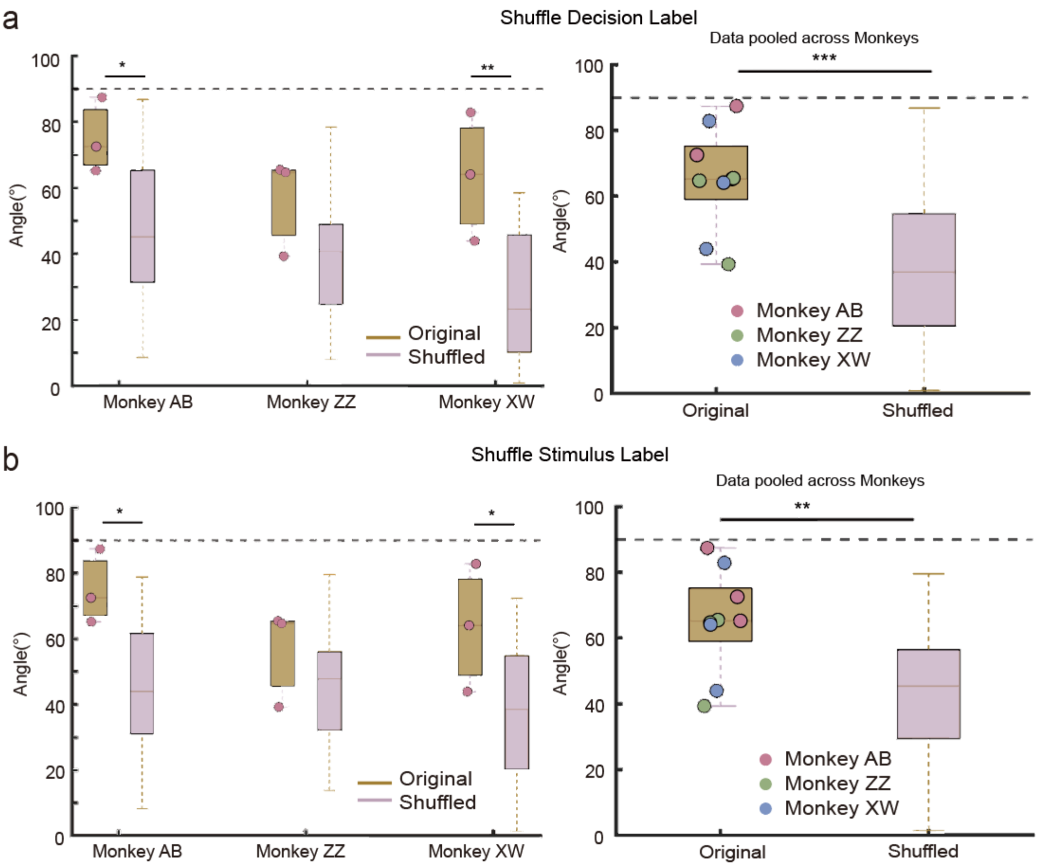

Supplementary Fig. 14. Subspace near-orthogonality validated through shuffle control analyses. (a) Left panel: Subspace angle between decision- and stimulus-related components after shuffling decision labels while preserving stimulus labels for individual monkeys. Right panel: Cross-animal statistics. (Original group: n = 3 sessions per monkey; Shuffled group: n = 30 shuffled iterations per monkey; Monkey AB: P = 0.03, g = 1.36; Monkey XW: P=0.003, g=1.94; combined Monkey: P = 0.0002, g = 1.31). (b) Left panel: Subspace angle after shuffling stimulus labels while preserving decision labels for individual monkeys. Right panel: Cross-animal statistics. Data are shown as box-and-whisker plots. The line within each box indicates the median; box edges represent the first (Q1) and third (Q3) quartiles; whiskers show the full data range. Colored scatter dots represent individual sessions, with each color corresponding to one monkey. (Original group: n = 3 sessions per monkey; Shuffled group: n = 30 shuffled iterations per monkey; Monkey AB: P = 0.01, g = 1.55; Monkey XW: P=0.04, g = 1.25; combined Monkey: P=0.001, g=1.14). *, $P < 0.05$, **, $P < 0.01$, ***, $P < 0.001$, NS, not significant, Watson–Williams test.

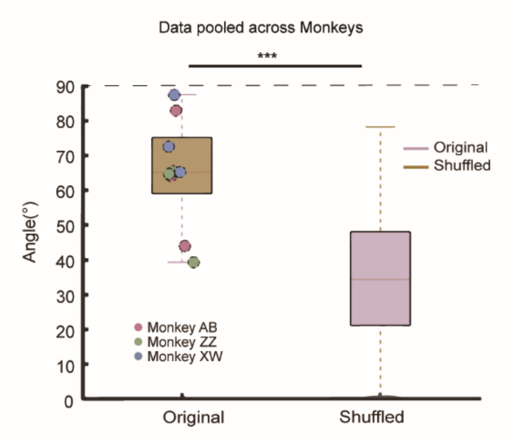

Supplementary Fig. 15. Near-orthogonal relationship between decision- and stimulus-related subspace. Angle between the stimulus- and decision-related subspaces combined across three monkeys, with black asterisks indicating comparisons with angles after shuffling decision and stimulus labels. Data are shown as box-and-whisker plots. The line within each box indicates the median; box edges represent the first (Q1) and third (Q3) quartiles; whiskers show the full data range. Colored scatter dots represent individual sessions, with each color corresponding to one monkey (Original group: 3 sessions per monkey, $n = 9$ sessions; Shuffled group: 30 shuffled iterations per monkey, $n = 90$; $P < 0.001$, $g = 1.66$). *, $P < 0.05$, **, $P < 0.01$, ***, $P < 0.001$, NS, not significant, Watson–Williams test.

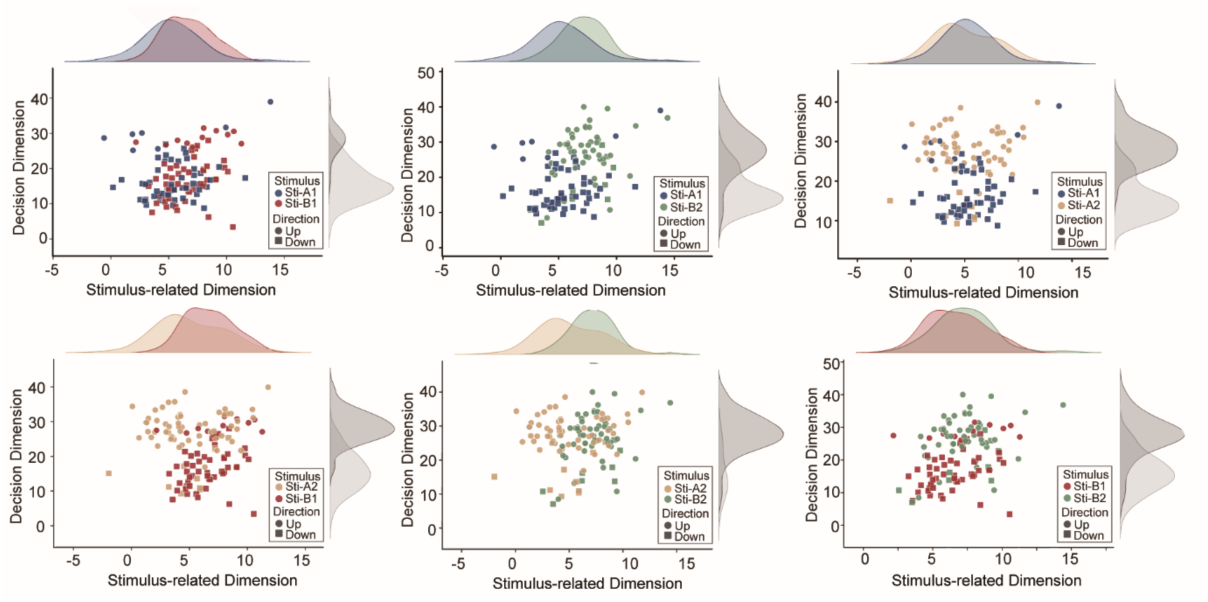

Supplementary Figure 16. Pairwise analysis of stimulus separability in stimulus-related dimension for Monkey AB. Each panel shows scatter plots and distributions of pairwise visual stimuli in both stimulus-related and decision dimensions. Data shown are from a representative session.

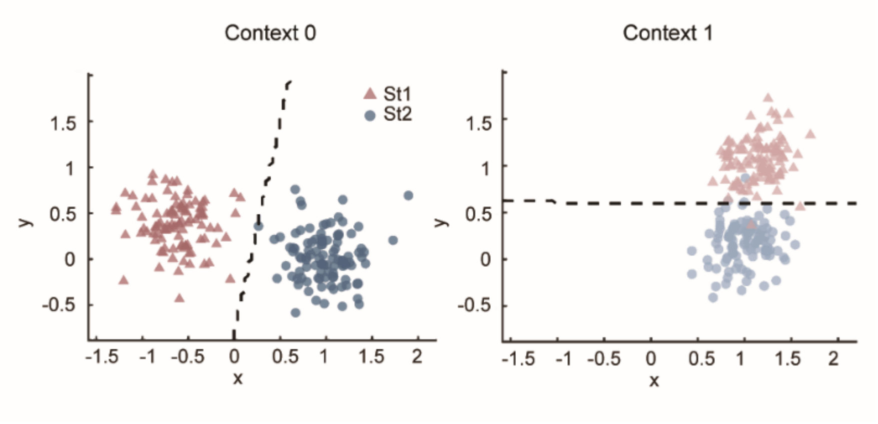

Supplementary Fig. 17. Linear toy model demonstrating separable but unstable dynamic encoding. Left: Two stimulus categories (blue circles and red triangles) are linearly separable in Context 0 under identity transformation. Right: The same stimulus categories remain separable in Context 1 after 10° rotation. Sti, Stimulus.

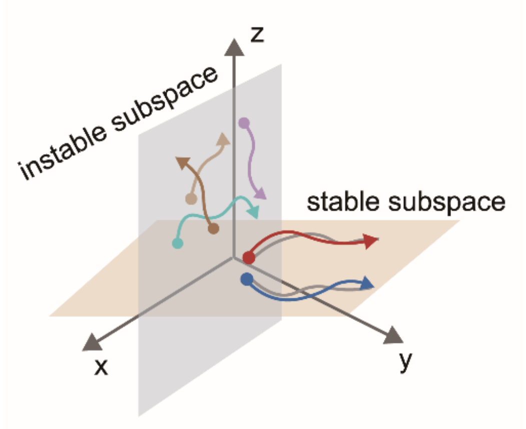

Supplementary Fig. 18. Example of near-orthogonality between stable and unstable subspaces. The stable subspace (yellow plane, xy-plane) maintains a fixed manifold structure, while the unstable subspace (gray plane, z-axis) can undergo arbitrary transformations. Despite dynamic changes in the unstable dimension, orthogonality with the stable subspace is preserved, demonstrating that instability within one subspace does not compromise its orthogonal relationship with another subspace.

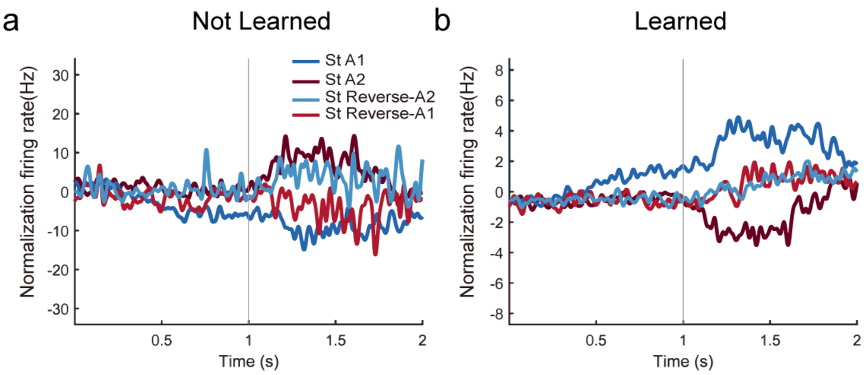

Supplementary Fig. 19. The decision representations in the reverse task for Monkey ZZ. Neural manifolds in the principal component of the decision subspace during Task A and Reverse-A. (a) Decision subspace manifolds between Task A and the first 20 trials of Reverse-A (not learned stage); (b) Decision subspace manifolds between Task A and Reverse-A after reaching the learning criterion, ≥90% correct over 15 consecutive trials (learned stage). Data shown are from one representative session. St, Stimulus.

References:

[1] Cunningham, J. P. & Yu, B. M. Dimensionality reduction for large-scale neural recordings. Nat. Neurosci. 17, 1500 – 1509 (2014).

[2] Zhang, C., Bengio, S., Hardt, M., Recht, B. & Vinyals, O. Understanding deep learning requires rethinking generalization. In Proc. Int. Conf. Learn. Represent. (ICLR, 2017).

[3] Golub, M. D. et al. Learning by neural reassociation. Nat. Neurosci. 21, 607 – 616 (2018).

[4] Pandarinath, C. et al. Inferring single-trial neural population dynamics using sequential auto-encoders. Nat. Methods 15, 805 – 815 (2018).

[5] Smith, E. H. et al. Widespread temporal coding of cognitive control in the human prefrontal cortex. Nat. Neurosci. 22, 1883–1891 (2019).

[6] Tan, H. E., Sisti, A. C., Jin, H. et al. The gut – brain axis mediates sugar preference. Nature 580, 511 – 516 (2020).

[7] Runyan, C. A., Piasini, E., Panzeri, S. & Harvey, C. D. Distinct timescales of population coding across cortex. Nature 548, 92–96 (2017).

[8] Drieu, C. et al. Rapid emergence of latent knowledge in the sensory cortex drives learning. Nature 2025, 1–11 (2025).

[9] Kobak, D., Brendel, W., Constantinidis, C., et al. Demixed principal component analysis of neural population data. eLife 5, e10989 (2016).

[10] Rutherford, A. Introducing ANOVA and ANCOVA: A GLM Approach (SAGE Publications, 2001).

[11] Bartlett, F. C. Remembering: A Study in Experimental and Social Psychology (Cambridge Univ. Press, 1932).

[12] Piaget, J. The Origins of Intelligence in Children (International Universities Press, 1952).

[13] Ghosh, V. E. & Gilboa, A. What is a memory schema? A historical perspective on current neuroscience literature. Neuropsychologia 53, 104 – 114 (2014).

[14] Zhou, J. et al. Evolving schema representations in orbitofrontal ensembles during learning. Nature 590, 606 – 611 (2021).

[15] Baraduc, P. et al. Schema cells in the macaque hippocampus. Science 363, 635 – 639 (2019).

[16] Warren, D. E. et al. False recall is reduced by damage to the ventromedial prefrontal cortex:

implications for understanding the neural correlates of schematic memory. J. Neurosci. 34, 7677 – 7682 (2014).

[17] Webb, C. E. et al. What's the gist? The influence of schemas on the neural correlates underlying true and false memories. Neuropsychologia 93, 61 – 74 (2016).

[18] Kizilirmak, J. M. et al. Neural correlates of learning from induced insight: a case for reward-based episodic encoding. Front. Psychol. 7, 1693 (2016).

[19] Pesaran, B., Nelson, M. J. & Andersen, R. A. Free choice activates a decision circuit between frontal and parietal cortex. Nature 453, 406 – 409 (2008).

[20] Hoshi, E. & Tanji, J. Distinctions between dorsal and ventral premotor areas: anatomical connectivity and functional properties. Curr. Opin. Neurobiol. 17, 234 – 242 (2007).

[21] Bobrowicz, K. & Greiff, S. Executive functions in birds. Birds 3, 184 – 220 (2022).

[22] Dhawan, S. S., Tait, D. S. & Brown, V. J. More rapid reversal learning following overtraining in the rat is evidence that behavioural and cognitive flexibility are dissociable. Behav. Brain Res. 363, 45 – 52 (2019).
