# Supplementary material for: Domain-specific schema reuse supports flexible learning to learn in the primate brain
Source: Nat Commun. 2026 Jan 29;17:2150. doi: 10.1038/s41467-026-68692-x (PMC12957312; doi:10.1038/s41467-026-68692-x)
Supplement: Supplementary file 2 — Reporting Summary [file 41467_2026_68692_MOESM2_ESM.pdf]

nature portfolio

Corresponding author(s): Shan Yu, Jianwen Gu

Last updated by author(s): Dec 12, 2025

Reporting Summary

Nature Portfolio wishes to improve the reproducibility of the work that we publish. This form provides structure for consistency and transparency in reporting. For further information on Nature Portfolio policies, see our Editorial Policies and the Editorial Policy Checklist.

Statistics

For all statistical analyses, confirm that the following items are present in the figure legend, table legend, main text, or Methods section.

n/a	Confirmed	
☐	☒	The exact sample size (*n*) for each experimental group/condition, given as a discrete number and unit of measurement
☐	☒	A statement on whether measurements were taken from distinct samples or whether the same sample was measured repeatedly
☐	☒	The statistical test(s) used AND whether they are one- or two-sided<br>*Only common tests should be described solely by name; describe more complex techniques in the Methods section.*
☐	☒	A description of all covariates tested
☐	☒	A description of any assumptions or corrections, such as tests of normality and adjustment for multiple comparisons
☐	☒	A full description of the statistical parameters including central tendency (e.g. means) or other basic estimates (e.g. regression coefficient) AND variation (e.g. standard deviation) or associated estimates of uncertainty (e.g. confidence intervals)
☐	☒	For null hypothesis testing, the test statistic (e.g. *F*, *t*, *r*) with confidence intervals, effect sizes, degrees of freedom and *P* value noted<br>*Give P values as exact values whenever suitable.*
☒	☐	For Bayesian analysis, information on the choice of priors and Markov chain Monte Carlo settings
☒	☐	For hierarchical and complex designs, identification of the appropriate level for tests and full reporting of outcomes
☐	☒	Estimates of effect sizes (e.g. Cohen's *d*, Pearson's *r*), indicating how they were calculated

Our web collection on statistics for biologists contains articles on many of the points above.

Software and code

Policy information about availability of computer code

Data collection	Cereplex Direct (Blackrock, USA), Monkeylogic
Data analysis	Offline Sorter v4, Mountainsort, Matalab R2023b, dPCA matlab toolbox, Python 2.7, Tensorflow, LFADS<br>The code used to analyze the data and generate the figures in this study is available on GitHub at https://github.com/kathytian-kx/DomainSpecific_SchemaReuse.git, and has been deposited in Zenodo (https://doi.org/10.5281/zenodo.17909108).

For manuscripts utilizing custom algorithms or software that are central to the research but not yet described in published literature, software must be made available to editors and reviewers. We strongly encourage code deposition in a community repository (e.g. GitHub). See the Nature Portfolio guidelines for submitting code & software for further information.

Data

Policy information about availability of data

All manuscripts must include a data availability statement. This statement should provide the following information, where applicable:

- Accession codes, unique identifiers, or web links for publicly available datasets
- A description of any restrictions on data availability
- For clinical datasets or third party data, please ensure that the statement adheres to our policy

The processed data used to generate results in this study are provided in the Source Data file. The raw electrophysiological data are available under restricted

access due to institutional policies. Access can be requested by contacting the corresponding author at shan.yu@nlpr.ia.ac.cn. Requests will be responded to within two weeks, and data will remain available for one year after access is granted. Source data are provided with this paper.

Research involving human participants, their data, or biological material

Policy information about studies with [human participants or human data](). See also policy information about [sex, gender (identity/presentation), and sexual orientation]() and [race, ethnicity and racism]().

Reporting on sex and gender	*Use the terms sex (biological attribute) and gender (shaped by social and cultural circumstances) carefully in order to avoid confusing both terms. Indicate if findings apply to only one sex or gender; describe whether sex and gender were considered in study design; whether sex and/or gender was determined based on self-reporting or assigned and methods used.*<br>*Provide in the source data disaggregated sex and gender data, where this information has been collected, and if consent has been obtained for sharing of individual-level data; provide overall numbers in this Reporting Summary. Please state if this information has not been collected.*<br>*Report sex- and gender-based analyses where performed, justify reasons for lack of sex- and gender-based analysis.*
Reporting on race, ethnicity, or other socially relevant groupings	*Please specify the socially constructed or socially relevant categorization variable(s) used in your manuscript and explain why they were used. Please note that such variables should not be used as proxies for other socially constructed/relevant variables (for example, race or ethnicity should not be used as a proxy for socioeconomic status).*<br>*Provide clear definitions of the relevant terms used, how they were provided (by the participants/respondents, the researchers, or third parties), and the method(s) used to classify people into the different categories (e.g. self-report, census or administrative data, social media data, etc.)*<br>*Please provide details about how you controlled for confounding variables in your analyses.*
Population characteristics	*Describe the covariate-relevant population characteristics of the human research participants (e.g. age, genotypic information, past and current diagnosis and treatment categories). If you filled out the behavioural & social sciences study design questions and have nothing to add here, write "See above."*
Recruitment	*Describe how participants were recruited. Outline any potential self-selection bias or other biases that may be present and how these are likely to impact results.*
Ethics oversight	*Identify the organization(s) that approved the study protocol.*

Note that full information on the approval of the study protocol must also be provided in the manuscript.

Field-specific reporting

Please select the one below that is the best fit for your research. If you are not sure, read the appropriate sections before making your selection.

☒ Life sciences ☐ Behavioural & social sciences ☐ Ecological, evolutionary & environmental sciences

For a reference copy of the document with all sections, see [nature.com/documents/nr-reporting-summary-flat.pdf]()

Life sciences study design

All studies must disclose on these points even when the disclosure is negative.

Sample size	Behavioral data were collected over multiple sessions: 5 days for Monkey AB (383.80±106.23 trials/day), 5 days for Monkey XW (225.40 ±33.22 trials/day), and 4 days for Monkey ZZ (390.25±105.83 trials/day). In total, 728 single- and multi-unit neural signals were recorded. Neural data analysis focused on three selected recording days per monkey. On these days, monkeys completed 420.67±129.08 (AB), 357.00 ±109.34 (ZZ), and 209.00±6.56 (XW) trials per day (mean±SD). Sample sizes were based on standard practice in non-human primate neurophysiology and ensured sufficient statistical power for within-subject comparisons. No statistical methods were used to predetermine sample size.
Data exclusions	Any session that presented issues with synchronization of recording hardware (neural, behavior), poor expression of spontaneous behaviors or large amounts of noise in the neural activity were excluded.
Replication	Behavioral and neural phenomena were examined across three monkeys (AB, XW, ZZ), each completing multiple sessions. Due to differences in task design across animals, the core findings shown in Figures 2 and 3 were consistently observed in all three monkeys. The results in Figures 4 and 5, which involved specific task variants, were reproducible in two monkeys. Key effects—including learning performance, schema reuse, and neural population dynamics—were verified using multiple analytical approaches (e.g., decoding, dimensionality reduction, and correlation analysis) to ensure robustness.
Randomization	The internal conditions of each task in the macaque experiment were all randomized.
Blinding	Blinding was applied during both data collection and analysis. Spike-sorting was completed prior to data analysis, blind to downstream analyses.

Reporting for specific materials, systems and methods

We require information from authors about some types of materials, experimental systems and methods used in many studies. Here, indicate whether each material, system or method listed is relevant to your study. If you are not sure if a list item applies to your research, read the appropriate section before selecting a response.

Materials & experimental systems

n/a	Involved in the study
☒	☐ Antibodies
☒	☐ Eukaryotic cell lines
☒	☐ Palaeontology and archaeology
☐	☒ Animals and other organisms
☒	☐ Clinical data
☒	☐ Dual use research of concern
☒	☐ Plants

Methods

n/a	Involved in the study
☒	☐ ChIP-seq
☒	☐ Flow cytometry
☒	☐ MRI-based neuroimaging

Animals and other research organisms

Policy information about studies involving animals; ARRIVE guidelines recommended for reporting animal research, and Sex and Gender in Research

Laboratory animals

Three adult male rhesus macaques (Macaca mulatta) were used in this study: Monkey AB (9.8 kg, 9 years old), Monkey ZZ (10.6 kg, 10 years old), and Monkey XW (8.9 kg, 6 years old). All animals were housed in individual primate cages under controlled conditions (temperature, humidity, and 12-h light/dark cycle), with regular access to environmental enrichment and full veterinary care. Experimental procedures were conducted in a dedicated primate neuroscience facility.

Wild animals

Not applicable.

Reporting on sex

Only male animals were used in this study. Sex-based differences were not analyzed.

Field-collected samples

Not applicable.

Ethics oversight

All procedures were approved by the Biomedical Research Ethics Review Committee of the Institute of Automation, Chinese Academy of Sciences (approval number: IA-201904), and complied with the National Institutes of Health Guide for the Care and Use of Laboratory Animals.

Note that full information on the approval of the study protocol must also be provided in the manuscript.

Plants

Seed stocks

Report on the source of all seed stocks or other plant material used. If applicable, state the seed stock centre and catalogue number. If plant specimens were collected from the field, describe the collection location, date and sampling procedures.

Novel plant genotypes

Describe the methods by which all novel plant genotypes were produced. This includes those generated by transgenic approaches, gene editing, chemical/radiation-based mutagenesis and hybridization. For transgenic lines, describe the transformation method, the number of independent lines analyzed and the generation upon which experiments were performed. For gene-edited lines, describe the editor used, the endogenous sequence targeted for editing, the targeting guide RNA sequence (if applicable) and how the editor was applied.

Authentication

Describe any authentication procedures for each seed stock used or novel genotype generated. Describe any experiments used to assess the effect of a mutation and, where applicable, how potential secondary effects (e.g. second site T-DNA insertions, mosiacism, off-target gene editing) were examined.
